# Stem Cells in Regenerative Medicine

**DOI:** 10.3390/jcm11185460

**Published:** 2022-09-16

**Authors:** Antonio Casado-Díaz

**Affiliations:** Unidad de Gestión Clínica de Endocrinología y Nutrición, Hospital Universitario Reina Sofía, Instituto Maimónides de Investigación Biomédica de Córdoba (IMIBIC), Centro de Investigación Biomédica en Red de Fragilidad y Envejecimiento Saludable (CIBERFES), 14004 Córdoba, Spain; bb1cadia@uco.es

Stem cells constitute a set of undifferentiated cells with the capacity to differentiate into other cell types and to self-renew. Stem cells can be: (i) totipotent, such as the ones of the zygote, which can give rise to any cell in an organism and to extraembryonic structures; (ii) pluripotent, such as embryonic cells that can differentiate into any cell in the germ layers; (iii) multipotent if they can differentiate into different cell types within specific lineages, such as hematopoietic stem cells; (iv) oligopotent, with the capacity to differentiate into only some cell types; and (v) unipotent, which gives rise to a specific cell type. In adults, there are multipotent, oligopotent and unipotent stem cells, being distributed throughout the organism. Their functions involve maintaining tissue homeostasis and regeneration. These include mesenchymal, hematopoietic, neural and dermal stem cells (MSC, HSC, NSC and DSC, respectively).

For ethical reasons, clinical applications of stem cells are developed mainly using adult stem cells. Fortunately, pluripotent stem cells can be obtained by genetic reprogramming of adult somatic cells. They are called induced pluripotent stem cells (iPSC). Among stem cells, HSCs have been studied for more than 50 years. Their isolations from different sources, such as bone marrow, peripheral blood or umbilical cord blood, have been described and standardized, as well as their use in cell therapy. HSCs are mainly used in transplantation, in patients with an inefficient hematopoietic system, which may be caused by pathologies, such as leukemia or anemia [1].

For their part, MSCs in the last 20 years have become the most studied stem cells, exhibiting the greatest potential for regenerative medicine applications. MSCs were identified in guinea-pig bone marrow and spleen by Friedenstein in 1970, being described as fibroblastic cells with the capacity to differentiate into osteoblasts [2]. Later, it was shown that MSCs can also differentiate into other cell types, derived from the mesoderm, such as adipocytes and chondrocytes. MSCs can even differentiate into cells of endodermal and ectodermal origin, such as hepatocytes and neurons.

MSCs can be obtained from different tissues, including bone marrow, fat tissue, umbilical cord, hair follicle, periodontal ligament and placenta. Once isolated, they must be expanded and characterized for possible therapeutic use. In order to homogenize the criteria defining MSCs, the International Society for Cell Therapy (ISCT) defined their minimum characteristics. Thus, they must: (i) be plastic adherent under standard culture conditions; (ii) express the surface markers CD-73, CD-90 and CD-105; and (iii) lack expression of CD-11b, CD-14, CD-19, CD-34, CD-45, CD-79a and HLADR. In addition, they must have the capacity to differentiate into osteoblasts, adipocytes or chondrocytes in vitro [3]. The main MSC characteristics that define their therapeutic potential are: (i) immunomodulatory activity; (ii) differentiation capacity; (iii) easy isolation from their source and further expansion in vitro; (iv) susceptible to cryopreservation; (v) hypoimmunogenic, expressing low levels of Major Histocompatibility Complex (MHC) class I and II; (vi) susceptible to intravenous administration; and (vii) involved in cell signaling, producing and secreting paracrine factors with regenerative capacity.

Numerous studies have demonstrated the immunomodulatory character of MSCs. Their application in the treatment of different pathologies has evidenced their capacity to regulate the immune response. They have been shown to suppress T-cell proliferation, cytokine secretion and cytotoxicity, regulating the Th1/Th2 balance and the functions of regulatory T cells (Tregs). They also increase B-cell viability and affect antibody secretion and the production of B-cell co-stimulatory molecules. Additionally, they inhibit the maturation and activation of dendritic cells and natural killer (NK) cells induced by interleukin-2 (IL-2). These actions are mediated by secreting immunosuppressive factors, such as TGF-β, PGE2 and IL-10 [4].

With regard to their differentiation capacity, MSCs can differentiate into different cell types in vitro, under the appropriate stimuli. Among them are osteoblasts, chondrocytes and adipocytes. This differentiation capacity has allowed the development of very interesting therapeutic strategies. For example, MSCs can be included in biocompatible scaffolds containing factors that induce their differentiation into the desired cell types. Different types of biomaterials have been experimented on for this purpose. They include bioceramics, polymers and composite biomaterials. They must be biocompatible, biodegradable and osteoinductive. Their structure must allow the three-dimensional growth of MSCs, as well as their communication with the tissues surrounding the implant. In relation to added factors, osteoinductive ones may include bone morphogenetic proteins (BMP), fibroblast growth factor (FGF), transforming growth factor-β1 (TGFB1) and vascular endothelial growth factor (VEGF). The latter is an important angiogenic factor. It can induce the formation of vessels that supply nutrients and oxygen to the scaffold, thus, ensuring survival of implanted MSCs. These strategies have been evaluated in numerous preclinical studies, as well as several clinical trials, with very promising results. For instance, they have been used in the treatment of difficult-to-heal bone fractures [5].

Implantation of MSCs in tissue-injured areas has shown that a high proportion of MSCs does not participate in tissue regeneration through cell differentiation. Rather, they participate in the creation of a suitable microenvironment, favoring proliferation and differentiation of endogenous cells. For this reason, much interest has been directed in recent years to the paracrine factors secreted by MSC. These factors constitute the MSC secretome, being composed of soluble ones and extracellular vesicles [6]. The former includes cytokines, chemokines, immuno-modulatory molecules and growth factors, such as TGF-β, PGE2, IL-10, VEGF, HGF, IGF and FGF. In relation to extracellular vesicles, exosomes of endosomal origin and between 30 and 150 nm in size stand out. They are composed of a lipid bilayer with different membrane proteins. Their cargos include nucleic acids, proteins and different metabolites. Indeed, exosomes are a vehicle of intercellular communication, involved in homeostasis and tissue regeneration, as well as in pathological processes. Extracellular vesicles secreted by MSCs can affect the composition of the extracellular matrix. That can be accomplished through matrix-remodeling enzymes or by cellular physiological processes, after interacting with their host cells. Thus, MSC-derived exosomes can intervene in processes related to tissue regeneration, such as proliferation, differentiation, apoptosis, angiogenesis, migration and immunomodulation, among others.

On the other hand, it is important to note that MSC culture and expansion for use in cell therapy are carried out outside their natural niche. Thus, such conditions may influence their regenerative capacity. Therefore, it is essential to develop suitable culture conditions that allow for their expansion, without impairing their therapeutic properties. The MSC manufacturing process for clinical use should comply with the principles of Good Manufacturing Practice (GMP). This ensures that cell preparations are produced, processed and stored with the necessary controls for clinical use [7]. It should be taken into account that in vitro expansion of MSCs can produce genetic instability in cells. That might favor their potential tumorigenicity when they are implanted in patients. There is also risk of infection during isolation and administration procedures. Other drawbacks include their putative pro-fibrogenic potential and lung entrapment. That is particularly relevant when they must pass through small capillaries. Other potential issues with MSCs in cell therapy include their heterogeneous differentiation ability of distinct MSC populations.

Fortunately, the use of MSC-derived exosomes can bypass some of the limitations of using MSCs for clinical purposes. Among the advantages of the former in regenerative medicine are: (i) can be easily stored; (ii) production of large quantities of cells is not necessary; (iii) can be evaluated for safety, dosage and activity, as conventional pharmaceutical agents; (iv) have a long half-life and stability; (v) can be more easily applied than proliferative cells in the clinic; (vi) can circulate through the smallest capillaries, crossing the blood–brain barrier; (vii) have lower risks of immune rejection, cellular dedifferentiation or tumor formation than cellular therapies; and (viii) can be manipulated for more precise effects as therapeutic agents [8]. Manipulation of their cargos can be accomplished by MSC preconditioning under certain conditions, favoring the secretion of desired factors; for example, conditions of hypoxia or inflammation, when the aim is to obtain vesicles enriched in angiogenic or immunomodulatory factors [9]. In addition, application of different biotechnological techniques on these vesicles can alter their composition, transforming them into drug-carrying vehicles, with great customized therapeutic potential.

In addition to the development of cell therapy or cell-free therapy, application of stem cells in regenerative medicine can also be performed using other strategies. They include inducing mobilization of the patient’s stem cells from their reservoirs, such as bone marrow, into damaged tissues [10]. Indeed, this process occurs naturally, upon the occurrence of an injury, such as a skin ulcer, bone fracture or myocardial infarction. One of the main factors involved in stem cell mobilization is SDF-1α. This soluble protein binds to its receptor CXCR4, located on the surface of the stem cell membrane. This way, it promotes cell migration into areas with higher concentrations of SDF-1α. Under physiological conditions, such a concentration is higher in bone marrow, which maintains progenitor cells in this niche, than in other tissues. Interestingly, upon injury, tissues secrete SDF-1α, increasing its concentration and, thus, reversing its previous gradient. In this way, progenitor cells, such as MSCs or endothelial progenitor cells (EPCs), are mobilized outside the bone marrow. Thus, they are directed into the damaged tissue, facilitating its regeneration [11].

On the other hand, there is evidence that aging and certain pathologies, such as diabetes, may decrease the capacity to mobilize progenitor cells. Therefore, therapeutic strategies that favor mobilization of these precursor cells can facilitate regenerative processes, such as, for example, healing chronic ulcers in diabetics [12]. Usually, these strategies are based on the use of drugs that induce stem cell mobilization. Among these compounds are granulocyte colony stimulant (G-CSF) and the CXCR4 antagonist, called plerixafor or AMD3100. It is used to mobilize progenitor cells in peripheral blood after bone marrow transplantation [13]. Recently, it has been shown that other drugs, such as teriparatide (PTH1-34) and DPP4 inhibitors (DPP4i), can favor progenitor-cell mobilization, being, therefore, proposed for regenerative medicine [14,15].

The possibility of using different therapeutic strategies based on stem cells is very promising for progress in regenerative medicine. Yet, there are still many questions to be answered in order to improve the efficiency of these treatments, before they can be widely used. In this scenario, there are numerous challenges to be addressed by basic and clinical research, in order to continue advancing in the development of new treatments, involving the use of stem cells. Among these challenges is the development of homogeneous, reproducible and efficient stem cell isolation, culture and expansion techniques. The same is applicable to the development of cell-free therapies, using extracellular vesicles. In this case, it is also essential to develop appropriate techniques that allow for their production, isolation and conservation, so that they can be used on a large scale in regenerative medicine.

In order to reach these challenges, this Special Issue about stem cells and regenerative medicine in the *Journal of Clinical Medicine* aims to be a meeting point for the latest advances in basic research, as well as application of stem cells and exosomes in regenerative medicine. Thus, contributing to such knowledge about stem cells and their derivatives should shed light on these exiting topics, which has the potential to improve clinical practice in a safer and more effective manner.
